# Exploring UK older adults’ dietary fibre consumption habits and associated factors: a national diet and nutrition survey perspective

**DOI:** 10.1017/S0007114524001557

**Published:** 2024-08-28

**Authors:** Victoria Norton, Yankho Kaimila, Julie A. Lovegrove, Stella Lignou

**Affiliations:** 1 Sensory Science Centre, Department of Food and Nutritional Sciences, Harry Nursten Building, University of Reading, Reading RG6 6DZ, UK; 2 Hugh Sinclair Unit of Human Nutrition, Department of Food and Nutritional Sciences, Harry Nursten Building, University of Reading, Reading RG6 6DZ, UK; 3 Institute of Cardiovascular and Metabolic Research, University of Reading, Reading RG6 6AA, UK; 4 Institute of Food, Nutrition and Health, University of Reading, Reading RG6 6EU, UK

**Keywords:** Dietary fibre, Older adults, Cross-sectional analysis, Oral health

## Abstract

The UK population is living longer; therefore, promoting healthy ageing via positive nutrition could have widespread public health implications. Moreover, dietary fibre intake is associated with health benefits; however, intake is below UK recommendations (30 g/d). Utilising national dietary survey data can provide up-to-date information on a large representative cohort of UK older adults, so that tailored solutions can be developed in the future. This study used cross-sectional data from the National Diet and Nutrition Survey (years 2008–2009 to 2018–2019) for older adults’ (*n* 1863; 65–96 years) dietary fibre intake (three-to-four-day food diaries), top ten dietary fibre-rich foods, associated factors (demographics, dietary/lifestyle habits) and various health outcomes (anthropometric, blood and urine). Mean dietary fibre intake was 18·3 g/d (range: 2·9–55·1 g/d); therefore, below the UK dietary recommendations, with compliance at 5·7 %. In addition, there were five significant associations (*P* < 0·05) related to lower dietary fibre intake such as increasing age group, without own natural teeth, impaired chewing ability, lower education leaving age and poor general health. Older adults’ key foods containing dietary fibre were mainly based on convenience such as baked beans, bread and potatoes. Positively, higher dietary fibre consumption was significantly associated (*P* = 0·007) with reduced diastolic blood pressure. In summary, the benefits of dietary fibre consumption were identified in terms of health outcomes and oral health were key modulators of intake. Future work should focus on a life course approach and the role of food reformulation to help increase dietary fibre intake.

There are approximately 12 million older adults (aged 65 years and above) in the UK population, with one in ten at risk of malnutrition or considered malnourished; therefore, promoting healthy ageing could provide widespread benefits at both an individual and population level^(1–3)^. Nutrition plays a key role in modulating health and well-being in the ageing population^(4)^. More specifically, dietary fibre-rich diets are associated with lower risk from CVD, coronary events, stroke, type 2 diabetes and colorectal cancer^(5)^. Sufficient dietary fibre intake can also improve bowel function, quality of life and instrumental activities of daily living as well as having beneficial associations with certain aspects of cognitive function (such as digit symbol substitution test score) and skeletal muscle mass in older adults^(6–11)^.

The dietary reference value for dietary fibre (quantitated by the Association of Analytical Chemists method) was increased to 30 g/d for UK adults (aged 19 years and over) in 2015^(5,12)^. However, adherence to dietary fibre recommendations in the UK and globally is low^(13–17)^. Therefore, it is important to put these values into context and understand how individuals can adhere to these 30 g/d recommendations^(18)^. There are a number of strategies that will increase dietary fibre intake such as a consumption of a variety of fruits and vegetables, wholegrain cereals and high dietary fibre snacks (e.g. nuts and seeds) to comply with the recommendations^(18,19)^. In the UK, dietary fibre intake in older adults is also well below the UK 30 g/d recommendations^(4,20)^. Kehoe *et al.* ^(7)^ reviewed older adults’ dietary fibre intake in eighteen countries from National Nutrition surveys and reported mean intakes of between 15–26 g/d, demonstrating that all countries surveyed were below the target from a UK guideline perspective. There is an urgent need to increase the dietary fibre intake in the population, particularly in older adults to promote public health.

Understanding barriers to dietary fibre consumption in older populations is key to successful dietary modulation and behavioural change. Food accessibility, preparation and consumption challenges, coupled with health status, age-related changes, environment, social support, income and nutrition knowledge can contribute to insufficient dietary fibre intake in an ageing population^(21,22)^. For example, oral health can be impacted by age often resulting in modulated food preferences favouring easy-to-chew foods; this may impact diet quality and variety as well as contribute to lower dietary fibre intake in an ageing population^(23–28)^. Accordingly, food structure is a key consideration in ensuring age-appropriate foods^(29)^. Unfortunately, dietary fibre-rich foods are often associated with negative textural attributes such as hard, fibrous, dry, sticky or adhesive, which are unlikely to improve or encourage consumption in an ageing population^(29)^. In addition, there are various barriers associated with encouraging dietary fibre intake which need to be addressed including cost, low awareness of health benefits, confusion around intake requirements, inadequate food package labelling, limited product choice, unappealing sensory profile, difficulty in consumption, negative terms associated with high dietary fibre intake (e.g. stodgy and bloating) and cooking constraints^(18,27,30–34)^. Going forward, overcoming such barriers will be key to promoting dietary fibre-rich diets.

Consideration of compliance to dietary fibre requirements is often overshadowed by other nutrients such as sugar, saturated fat and salt, highlighting the need for greater awareness and research into promoting dietary fibre intake, especially in an ageing population. This paper aims to understand (1) what proportion of the ageing UK population meets dietary fibre intake recommendations; (2) what dietary fibre-rich foods are typically consumed by older adults; and (3) what factors are associated with consuming dietary fibre-rich foods in UK older adults, by using the UK National Diet and Nutrition Survey (NDNS) (years 2008–2009 to 2018–2019) data, which includes a large representative cohort of UK older adults. These data will inform future strategies to enable an increase in dietary fibre intake within an older-aged population.

## Methods

### National diet and nutrition survey

The NDNS is an ongoing national cross-sectional survey focusing on food consumption, nutrient intake and nutritional status in a representative UK population (aged 1·5 years and above) based on private households^(16)^. In brief, invited individuals were selected at random (via the postcode address file) to ensure the data were a representative cohort of the UK population. The survey data collection centred around three aspects: (1) demographic and lifestyle questionnaires; (2) three-to-four-day unweighted food diary; and (3) obtaining physical measures of anthropometric outcomes, blood and urine samples^(16)^. All individuals provided informed consent and it was ensured that data would be collected in accordance with relevant data protection legislation as well as confidentiality being maintained at all times^(16)^. Ethical approval was granted from the corresponding ethical committees^(16)^.

### Study population

Adults aged 65 years and over (commonly referred to as older adults in the UK and globally) were selected for analysis^(35)^. Using years 2008–2009 to 2018–2019 from the NDNS resulted in a sample size of 1863 older adults (age range: 65–96 years; 42 % male and 58 % female).

### Dietary, anthropometric and biochemical measures

In brief, individuals were suitably trained and asked to complete an unweighted three-to-four-day food diary to record all food and drink consumed (including time, company, eating location, food and drink description, brand names, portion sizes and dishes consumed at home and eating out)^(16)^. The data were subsequently coded and integrated into the Diet In Nutrients Out system, and analysis was based on the NDNS Nutrient Databank^(16)^. The following measurements: height, weight, spot urine sample, blood pressure, non-fasting/fasting blood sample were also collected by a qualified nurse using validated approaches^(16)^. For the purpose of this paper, the focus was on dietary fibre which was based on the Association of Analytical Chemists method^(16)^. It should be noted some survey years have been retrospectively adjusted so that all data were reported using the Association of Analytical Chemists method^(16)^.

In addition, factors that can modulate food intake such as demographic characteristics, lifestyle preferences and dietary habits were captured during the initial interview stage^(16)^. Individuals’ height and weight were also recorded so that BMI was subsequently calculated^(16)^. Potential factors that older adults may encounter in terms of consuming sufficient dietary fibre intake were explored: (1) household income (less than £5000 to more than £100 000); (2) ethnicity (white, mixed ethnic group, black or black British, Asian or Asian British or any other group); (3) residence region (England: North, South, Central/Midlands, Wales Scotland and Northern Ireland); (4) survey year (2008–2009 to 2018–2019); (5) fruit and vegetable shopping frequency (more than once a day to less often than every two months); (6) fruit and vegetable shopping location (large supermarket to spontaneous); (7) food avoidance (yes *v*. no); (8) overall health (very bad to very good); (9) medication use (yes *v*. no); (10) own natural teeth (yes *v*. no); (11) denture use (yes *v*. no); (12) smoking (yes *v*. no); (13) chewing ability (no difficulty to great amount of difficulty); (14) education leaving age (14 years or under to 19 years or over); (15) food type (soft/mashed foods to liquids); and (16) health outcomes (physical activity level, BMI, systolic blood pressure, diastolic blood pressure, low-density lipoprotein cholesterol, high-density lipoprotein cholesterol, TAG, total cholesterol and glucose) were all assessed and added as covariates in the analysis.

### Statistical analysis

The statistical analysis was performed using Stata software (version 17·0, StataCorp LP, USA). The data were analysed as a survey; therefore, sampling weights (e.g. reduced sampling-related bias by taking into account the numbers of individuals within each strata) for each year of analysis (2008–2009 to 2018–2019) were applied^(36)^. The individual-level sampling weights (as recommended by NDNS) were applied for the analysis on the association of dietary fibre consumption with demographic characteristics and types of foods consumed as well as the association of dietary fibre intake with anthropometric measures and health outcomes^(16)^. The data are presented as means with se. It should be noted that for some variables (such as physical activity, BMI, blood pressure (systolic and diastolic) and blood analytes), data were not available for all 1863 older adults. Accordingly, this was adjusted in the analysis (by removing the observations with missing values) using the ‘SVY command’ in Stata^(36)^. In addition, linear regression was used to assess the association of dietary fibre consumption with demographic characteristics and health outcomes. More specifically, the analysis was conducted using three models: (1) model one compared the outcomes of interest (fruit and vegetable shopping frequency and location, food avoidance, overall health, medication use, own natural teeth, denture use, chewing ability, education leaving age and food type) with dietary fibre and analysis was controlled for energy (bivariate analysis); (2) model two compared outcomes of interest in model one with age and sex added as covariates (multivariate analysis); and (3) model three was an analysis of model two with additional covariates (household income, residence region, survey year and ethnicity) (multivariate analysis). The health outcomes of interest (physical activity, BMI, systolic blood pressure, diastolic blood pressure, low-density lipoprotein cholesterol, high-density lipoprotein cholesterol, TAG, total cholesterol and glucose) utilised the same three models described above in our regression analysis. For all analyses *P* < 0·05 was considered statistically significant.

## Results

**Fig. 1.**
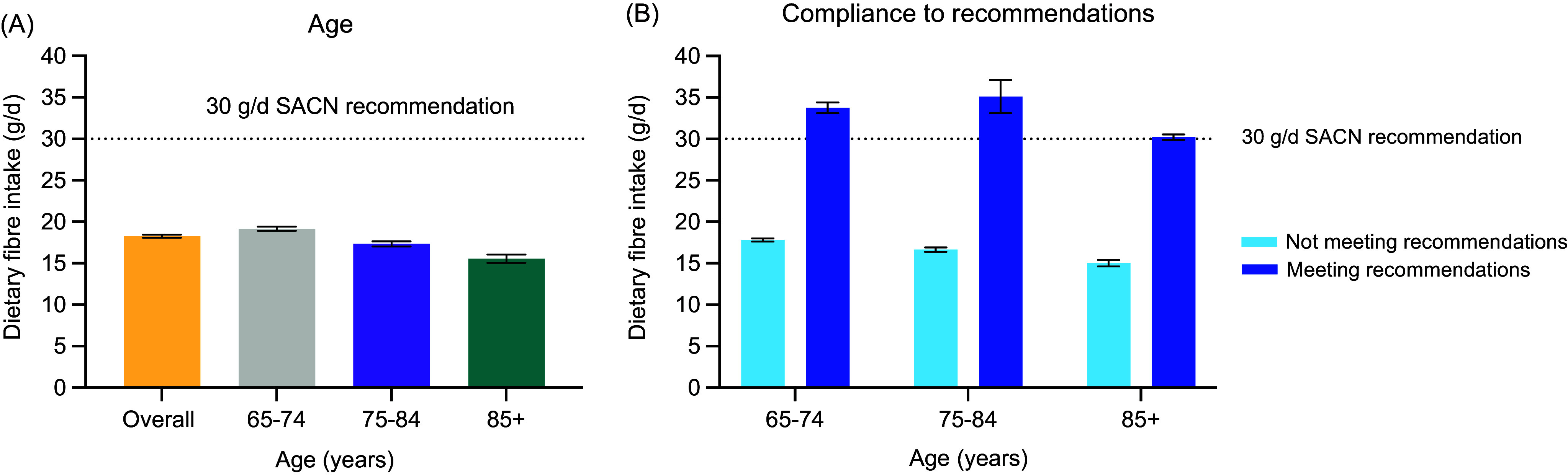
Older adults’ (*n* 1863) mean (± se) dietary fibre intake by (**A**) age: all age groups combined (overall) and increasing age (years; 65–74: *n* 1063; 75–84: *n* 615 and 85–96: *n* 185) and (**B**) recommendation compliance: not meeting recommendations (*n* 1756; 65–74 years: *n* 983; 75–84 years: *n* 592; and 85–96 years: *n* 181) and meeting recommendations (*n* 107; 65–74 years: *n* 80; 75–84 years: *n* 23; and 85–96 years: *n* 4). SACN refers to Scientific Advisory Committee on Nutrition.

The majority of the older adult cohort were aged between 65–74 years (57·1 %), resident in England (59·4 %), white British (97·4 %) and typically shopping for fruit/vegetables at large supermarkets (76·9 %) (Table S1). Older adults’ mean consumption of dietary fibre was 18·3 g/d (range: 2·9–55·1 g/d); only 5·7 % of this population (*n* 107/1863) adhered to the current UK recommendations utilising average dietary fibre intake over a three-to-four-day period (Fig. 1). There was a significant association (*P* = 0·02) for age group, with increasing age resulting in lower dietary fibre consumption (multivariate: –0·78 ± 0·33). The extent of sex-related associations was also modulated by analysis. Males consumed significantly more dietary fibre than females in the bivariate analysis (*P* = 0·01); however, this significant association was reduced in the multivariate analysis (Table 1).

**Table 1.** Mean (± s e) dietary fibre intake in older adults (*n* 1863) and corresponding analysis by various factors

Factors	Variables	Total		Mean	se (g)	95 % CI		Multivariate analysis		
		*n*	%			Lower	Upper	Coefficient	se	*P*-value
Sex	Male	781	41·9	19·61	0·28	19·06	20·15	0·74	0·48	0·13
	Female	1082	58·1	17·20	0·25	16·70	17·70			
Fruit & vegetable shopping frequency	More than daily	8	0·4	18·58	2·70	13·29	23·87	–0·35	0·29	0·23
	Once a day	41	2·2	20·16	1·08	18·04	22·27			
	2 or 3 times a week	788	42·3	18·88	0·31	18·27	19·50			
	Weekly	911	48·9	17·71	0·26	17·21	18·21			
	2 or 3 times a month	76	4·1	18·08	0·98	16·15	20·01			
	Monthly	15	0·8	15·10	1·30	12·54	17·66			
	Every 2 months	5	0·3	17·79	0·96	15·90	19·68			
	Less often than 2 months	19	1·0	13·33	1·62	10·14	16·52			
Food avoidance*	Yes	914	49·1	18·54	0·29	18·0	19·1	–0·70	0·49	0·15
	No	948	50·9	18·02	0·26	17·5	18·5			
General health	Very good	431	23·1	19·98	0·39	19·22	20·74	–0·76	0·24	**0·001**
	Good	774	41·6	18·49	0·25	18·00	18·97			
	Fair	507	27·2	16·83	0·36	16·13	17·52			
	Bad	117	6·3	16·69	1·34	14·06	19·32			
	Very bad	34	1·8	16·19	1·22	13·79	19·59			
Medication*	Yes	1158	62·2	18·55	0·24	18·07	19·02	0·50	0·41	0·22
	No	202	10·8	20·73	0·52	19·72	21·75			
Own teeth	Yes	1405	75·4	19·00	0·22	18·52	19·40	–2·27	0·52	**< 0·0001**
	No	458	24·6	15·72	0·31	15·10	16·34			
Denture use	Yes	962	51·6	17·15	0·24	16·67	17·63	0·83	0·48	0·08
	No	901	48·4	19·28	0·28	18·72	19·83			
Smoking*	Yes	994	53·4	17·86	0·24	17·39	18·34	–0·19	0·21	0·37
	No	166	8·9	19·02	0·51	18·02	20·03			
Chewing ability*	No difficulty	1437	77·1	18·59	0·21	18·17	19·01	–1·32	0·45	**0·003**
	Little difficulty	329	17·6	17·49	0·49	16·52	18·46			
	Fair amount difficulty	78	4·2	16·20	1·08	14·08	18·32			
	Great amount difficulty	18	1·0	13·35	1·23	10·93	15·77			
Education age	Never went to school	5	0·3	16·48	3·28	10·04	22·91	0·58	0·14	**< 0·0001**
	14 or under	342	18·4	15·88	0·38	15·12	16·62			
	15	635	34·1	17·27	0·30	16·69	17·85			
	16	359	19·3	18·72	0·42	17·90	19·55			
	17	143	7·7	18·87	0·59	17·71	20·02			
	18	93	5·0	19·50	0·74	18·04	20·95			
	19 or over	265	14·2	21·64	0·54	20·58	22·71			
Food type*	Soft/mashed foods (only)	21	1·1	17·99	2·27	13·52	22·45	–0·93	0·29	**0·001**
	Other foods as well	394	21·2	16·94	0·46	16·04	17·84			
	Liquids (only)	3	0·2	21·45	3·29	15·00	27·90			

* Reflects missing data and/or not applicable: avoid food (*n* 1); medication (*n* 503); smoking (*n* 703); chewing ability (*n* 1); education age (*n* 21) and food type (*n* 1445; only a sub-group, therefore, may not be totally representative).

There was a significant association (*P* < 0·0001) with dentition, where older adults with their own natural teeth consumed more dietary fibre than individuals without their own natural teeth (Table 1). Similarly, older adults that reported impaired chewing ability had significantly lower dietary fibre intake (*P* = 0·003) compared with individuals with no chewing difficulty (Table 1). There was also a tendency for an association (*P* = 0·08) with denture use, with those who used dentures consuming less dietary fibre compared with non-denture users. The education leaving age had a significant impact on dietary fibre intake (*P* < 0·0001) where finishing school at a higher age was associated with increased dietary fibre intake. General health status was significantly associated (*P* = 0·001) with dietary fibre consumption where older adults that reported poorer general health had significantly lower dietary fibre intake. The remaining factors (such as household income, medication, smoking, fruit/vegetable shopping frequency and food avoidance) had no significant association with dietary fibre intake. However, it should be noted that all factors (apart from fruit and vegetable shopping location; *P* = 0·30) resulted in a significant association in the bivariate analysis; hence, included in subsequent multivariate analysis.

**Fig. 2.**
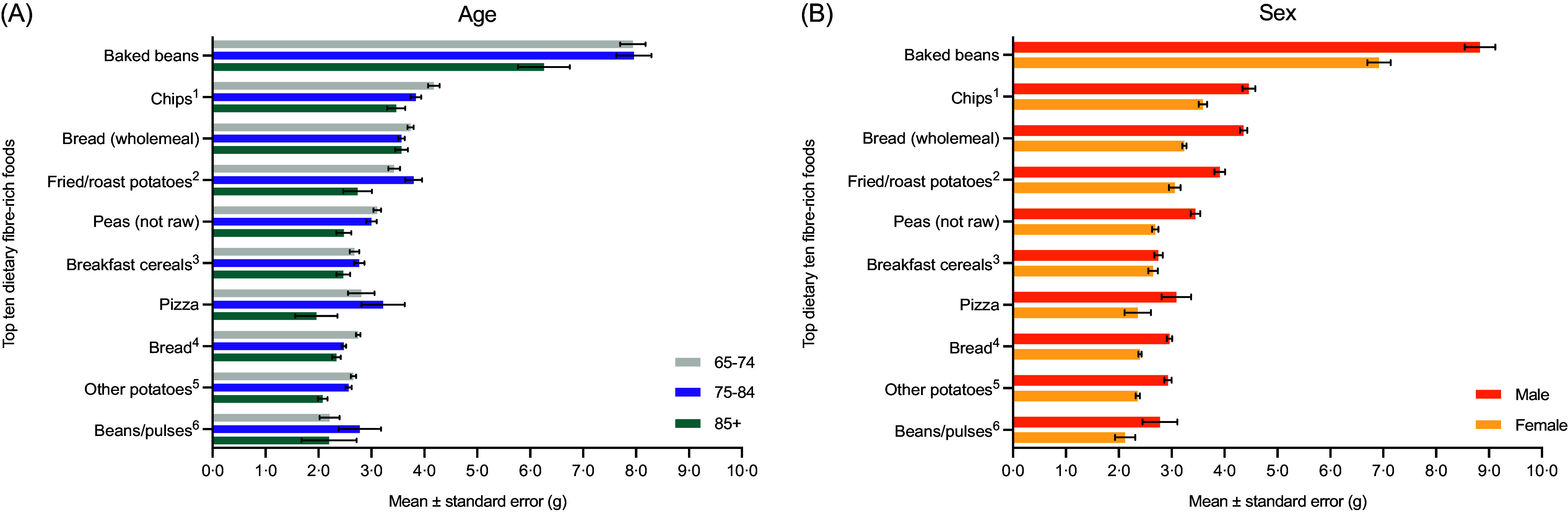
Older adults’ (*n* 1863) top ten dietary fibre-rich (mean ± se) by (**A**) age: (years; 65–74: *n* 1063; 75–84: *n* 615 and 85–96: *n* 185) and (**B**) sex (male = 781 and female: *n* 1082). Subscript number highlights additional information on food such as chips^1^: purchased including takeaway; fried/roast potatoes^2^: including homemade dishes; breakfast cereals^3^: wholegrain; and high fibre; bread^4^: brown, granary and wheatgerm; other potatoes^5^: including homemade dishes; and beans/pulses^6^: including ready meal and homemade dishes.

Older adults consumed the largest quantity of dietary fibre from baked beans, regardless of age and sex (Table 2 and Fig. 2). Overall, bread and potatoes were the most frequently consumed dietary fibre-rich foods within this population (Table 2). In addition, mean fruit and vegetable consumption was 121·1 g/d (range: 0·0–738·3 g/d) and just 24 % of older adults (*n* 448/1863) met the five-a-day recommendations.

**Table 2.** Top ten foods containing dietary fibre (mean ± se) consumed by older adults (*n* 1863)

Food	Totals*			Mean	se (g)	95 % CI	
	*n*	%	Obs			Lower	Upper
Baked beans	407	21·8	509	7·82	0·18	7·46	8·18
Chips^1^	555	29·8	710	4·02	0·07	3·88	4·17
Bread (wholemeal)	728	39·1	2817	3·66	0·04	3·59	3·74
Fried/roast potatoes^2^	526	28·2	839	3·49	0·09	3·32	3·66
Peas (not raw)	834	44·8	1185	3·03	0·05	2·92	3·13
Breakfast cereals^3^	297	15·9	999	2·69	0·61	2·57	2·81
Pizza	110	5·9	201	2·74	0·19	2·37	3·12
Bread (brown)^4^	659	35·4	2207	2·64	0·03	2·58	2·69
Other potatoes^5^	1078	57·9	2328	2·57	0·04	2·50	2·65
Beans/pulses^6^	159	8·5	566	2·33	0·16	2·01	2·65

* Totals: *n* and % relate to the number/percentage respectively of older adults consuming the specific food and Obs denotes observation (e.g. number of times this food reported in analysis). Subscript number highlights additional information on food such as chips^1^: purchased including takeaway; fried/roast potatoes^2^: including homemade dishes; breakfast cereals^3^: wholegrain; and high fibre; bread (brown)^4^: including granary and wheatgerm; other potatoes^5^: including homemade dishes; and beans/pulses^6^: including ready meal and homemade dishes.

BMI was significantly associated with dietary fibre intake (bivariate: 0·04 ± 0·01; *P* = 0·003) where an increase in BMI resulted in higher dietary fibre consumption; however, this association was reduced in the multivariate analysis (multivariate: 0·003 ± 0·03; *P* = 0·19). This was also the case for high-density lipoprotein cholesterol (bivariate: 1·05 ± 0·43; *P* = 0·02 *v*. multivariate: 0·74 ± 0·74; *P* = 0·32). Similarly, higher dietary fibre intake was significantly associated (bivariate: –0·69 ± 0·36; *P* = 0·05) with lower TAG; yet this effect was lost after multivariate analysis (model three: −0·89 ± 0·63; *P* = 0·16). It should be noted that only diastolic blood pressure was significantly associated (*P* = 0·007) with dietary fibre intake in all analyses; higher dietary fibre intake was associated with a lower diastolic blood pressure (–0·08 ± 0·03). Due to the lack of significant association between physical activity level, systolic blood pressure, low-density lipoprotein cholesterol, total cholesterol and glucose in the bivariate analysis, these outcomes were not included in subsequent analysis.

## Discussion

The mean dietary fibre intake of UK older adults was 18·3 g/d (range: 2·9–55·1 g/d) which was only 61 % of the current UK dietary reference value. This finding was not unexpected since data from eighteen European countries National Nutrition surveys also identified older adults’ dietary fibre intake was typically below target (15–26 g/d)^(7)^. However, Kehoe *et al.* ^(7)^ did not split older adults by increasing age groups; accordingly, age-related differences in intake within an ageing population may have been overlooked. Therefore, to address such a limitation our analyses grouped older adults into three groups: (1) young-old (aged 65–74 years); (2) old-old (aged 75–84 years); and (3) oldest-old (aged 85+ years)^(37,38)^. It was evident that our older adult cohort (75+ years) consumed less dietary fibre than their younger counterparts (65–74 years); this suggests the importance of recruiting sufficient older adults across the different age groups especially as the impact of age-related changes are likely to be greater with increasing age^(39–41)^. Moreover, it is important to understand adherence to guidelines within the population; our analysis demonstrated that only 107 from 1863 older adults adhered to current guidelines (using average dietary fibre intake over three-to-four-day period). However, it should be noted this is similar in the general adult population (19–64 years) for dietary fibre. For example, Scheelbeek *et al.* ^(15)^ noted approximately 7 % of the UK population met dietary fibre-related guidelines (Eatwell Guide). In addition, modelling related studies have demonstrated a small increase in dietary fibre intake (2·2 g/d) due to dietary fibre enrichment which could have widespread public implications^(42)^. Additionally, it is useful to investigate the role of various demographics on subsequent dietary fibre intake. Overall, basic demographics (such as household income, residence region, ethnicity and survey year) had no significant impact on dietary fibre consumption in this older adult cohort. Furthermore, a recent review highlighted the impact of diversity (e.g. background and health conditions) within this age group; this could be a possible reason for inconsistency in reporting associations between such demographics compared with other age groups as well as methodology differences in grouping individuals^(43)^. However, it should be noted that education leaving age had a noteworthy effect on dietary fibre. For example, higher education achievement resulted in higher dietary fibre intake; similar findings have been present in the literature where lower education led to lower dietary fibre intake and diet quality^(44,45)^. Therefore, this suggests more emphasis on a life course approach to dietary fibre and the role of food reformulation to help increase dietary fibre intake.

It is important to understand the relevant challenges associated with older adults’ dietary fibre intake so that targeted strategies to promote intake can be subsequently identified. For example, oral health can decline with age (such as teeth loss, chewing problems and lower bite force); thereby, negatively impacting dietary fibre intake (e.g. modulating food preferences and cooking preparation) within an ageing population^(23–28)^. Such findings were evident in our older adult cohort where individuals with poor chewing ability and without their own natural teeth tended to consume less dietary fibre. This suggests older adults with oral health impairments food preferences and diet quality are also influenced implying tailored advice with relevant substitutions would be helpful for this population^(23,46,47)^. Therefore, developing targeted strategies to meet older adults’ needs (including understanding relevant changes in oral processing behaviours with increasing age) would be suggested to overcome associated barriers and subsequently help reduce the widespread deficit in dietary fibre intake.

The top ten foods that contributed to older adults’ dietary fibre were compiled so that trends could be identified and areas of focus highlighted. The key dietary fibre-rich foods in terms of largest amount of dietary fibre and most commonly consumed were baked beans, bread and potatoes; suggesting convenience and foods requiring minimal effort was a key driver of food choice in this population. It is likely age-related changes could be a contributing factor resulting in preference for simple, easy/quick to prepare and convenience foods^(22)^. Moreover, Nakano and Washizu^(48)^ noted the shift in perceptions of convenience foods, and this could have a role in supporting healthy eating in an ageing population. In addition, convenience and easy access to food are considered to promote food intake and a key motivator in food choice; accordingly, this emphasises the importance of positive environmental cues on subsequent intake^(49,50)^. Going forward, improving fruit and vegetable consumption in this population would be recommended; in addition, this should have a noteworthy impact on dietary fibre consumption. However, it is not without its challenges such as concerns over food wastage (e.g. vegetable packet size too large in some cases), requiring regular shopping, cost implications, cooking skills and living alone^(22)^. Accordingly, this indicates the importance of making dietary fibre-rich foods accessible to all to reduce consumption-related barriers.

The health benefits of dietary fibre consumption are well-recognised such as reduction in CVD, coronary events, stroke, type 2 diabetes and colorectal cancer risk^(5)^. However, it is important to put such findings into context by investigating various health outcomes within our older adult cohort. General health (via self-reported measures) was related with higher dietary fibre intake as such highlighting that individuals identifying as healthier consumed more dietary fibre and clearly demonstrating the benefits of dietary fibre consumption. Moreover, it is important to understand whether perceived health can be explained by differences in relevant health markers. Most anthropometric and biochemical measures including BMI, physical activity level, systolic blood pressure, high-density lipoprotein cholesterol, low-density lipoprotein cholesterol, total cholesterol, TAG and glucose were not significantly associated with dietary fibre intake. However, there was a beneficial association between diastolic blood pressure and dietary fibre intake, where higher dietary fibre intake was associated with a lower diastolic blood pressure. If an older adult meets the dietary fibre 30 g/d recommendations, this was associated with a 2·5 mmHg lower diastolic blood pressure, which was clinically significant on a population scale. Cook *et al.* ^(51)^ noted that a 2·0 mmHg reduction in diastolic blood pressure could decrease hypertension prevalence as well as CHD and stroke risk. Therefore, developing easy to implement and accessible dietary fibre-related strategies for all is a key going forward.

The strength of the analysis is that the NDNS data include a representative cohort of UK older adults. However, it should be noted that such analysis is associated with various methodology challenges and potential limitations: (1) NDNS is a cross-sectional survey and identifies associations rather than causation and (2) potential bias may be linked with self-reported dietary intakes and potential underreporting^(52)^. In addition, the older adult cohort was mainly between 65–74 years old and 65 % of the cohort rated their general health either as good or very good. Therefore, going forward capturing adults in the oldest age group (85+ years) and more valuable health categories as well as underrepresented ethnic groups could provide additional age-related insights as dietary fibre intake may differ in such groups.

### Conclusion

This cross-sectional analysis observed that older adults’ dietary fibre intake was substantially below UK recommendations. Five key contributing factors (increasing age group, without own natural teeth, impaired chewing ability, lower education leaving age and poor general health) had a notable impact on subsequent dietary fibre intake. In addition, the role of easy-to-eat foods in older adults’ food choice should not be underestimated as baked beans, bread and potatoes were identified as key foods containing dietary fibre. Accordingly, promoting fruit and vegetables could lead to an uplift in dietary fibre consumption; however, any suggested foods also need to be tailored to the needs of this population (such as oral health impairments). Moreover, the beneficial association between health and dietary fibre (such as reduction in diastolic blood pressure and self-reported good general health) is noteworthy. This highlights the importance of increasing dietary fibre prior to entering the ‘older adults’ stage should be a key public health priority.
